# Thermomechanical Response of Polycarbonate/Aluminum Nitride Nanocomposites in Material Extrusion Additive Manufacturing

**DOI:** 10.3390/ma15248806

**Published:** 2022-12-09

**Authors:** Nectarios Vidakis, Markos Petousis, Panagiotis Mangelis, Emmanuel Maravelakis, Nikolaos Mountakis, Vassilis Papadakis, Maria Neonaki, Georgia Thomadaki

**Affiliations:** 1 Department of Mechanical Engineering, Hellenic Mediterranean University, 71410 Heraklion, Greece; 2 Department of Electronic Engineering, Hellenic Mediterranean University (HMU), 73133 Chania, Greece; 3 Institute of Molecular Biology and Biotechnology, Foundation for Research and Technology-Hellas, 71110 Heraklion, Greece

**Keywords:** three-dimensional (3D) printing, additive manufacturing, nanocomposites, polycarbonate (PC), aluminum nitride (AlN), material extrusion (MEX), mechanical characterization

## Abstract

Polycarbonate-based nanocomposites were developed herein through a material extrusion (MEX) additive manufacturing (AM) process. The fabrication of the final nanocomposite specimens was achieved by implementing the fused filament fabrication (FFF) 3D printing process. The impact of aluminum nitride (AlN) nanoparticles on the thermal and mechanical behavior of the polycarbonate (PC) matrix was investigated thoroughly for the fabricated nanocomposites, carrying out a range of thermomechanical tests. Scanning electron microscopy (SEM) and atomic force microscopy (AFM) provided information about the morphological and surface characteristics of the produced specimens. Using energy dispersive spectroscopy (EDS), the elemental composition of the nanocomposite materials was validated. Raman spectroscopy revealed no chemical interactions between the two material phases. The results showed the reinforcement of most mechanical properties with the addition of the AlN nanoparticles. The nanocomposite with 2 wt.% filler concentration exhibited the best mechanical performance overall, with the highest improvements observed for the tensile strength and toughness of the fabricated specimens, with a percentage of 32.8% and 51.6%, respectively, compared with the pure polymer. The successful AM of PC/AlN nanocomposites with the MEX process is a new paradigm, which expands 3D printing technology and opens a new route for the development of nanocomposite materials with multifunctional properties for industrial applications.

## 1. Introduction

PC nanocomposites have become highly attractive in recent years due to their superior mechanical and physical properties, which enable them to be used in a wide range of different applications. They have been investigated for the fabrication of membranes [1], light-weight building integrated photovoltaics [2], photodetector devices providing highly transparent and flexible substrates [3], coatings for biomedical engineering applications [4], and fire-safety evaluation systems [5]. PCs are a group of well-known thermoplastic polymers that exhibit good mechanical and aging performance, combining excellent optical and thermal properties [6,7].

The inclusion of additives in nanoscale form (particles, tubes, flakes) in a matrix material is a well-established strategy, with the aim of improving a wide range of material properties, such as mechanical, optical, electrical, and thermal, and optimizing the performance of the matrix material for various applications [8,9,10]. Many reports have shown that the addition of appropriate fillers in the PC polymer and the development of nanocomposites can effectively reinforce the mechanical response of the pure material [11,12,13], but also can improve the thermal stability, flammability [5], and optical properties [14]. For instance, the development of PC nanocomposites with silica nanoparticles showed improvements in both the mechanical and thermal properties of the materials, simultaneously increasing the Young’s modulus, yield strength, and thermal stability [15]. By adding small amounts of TiC or cellulose nanofibers to pure PC, remarkable reinforcements of mechanical performance have been achieved [16]. PC nanocomposite filaments infused with nanosilica at small concentrations up to 1 wt.% exhibited improved mechanical and optical properties, which are quite promising for thin film and automotive industry applications [14]. Films made of silver nanowire/polycarbonate nanocomposites were fabricated, exhibiting a high increase in thermal stability in combination with effective electrical conductivity, as well as a high optical transparency [17].

Many studies have revealed that nitride compounds are excellent fillers, and their mixture with appropriate polymers can greatly enhance their mechanical behavior and thermal and dielectric properties [18]. By adding boron nitride nanotubes to the PC polymer, the thermal stability of the nanocomposites was increased by 4–8 degrees, while the tensile toughness was simultaneously increased by 39% [19]. Moreover, the Young’s modulus was reinforced up to 31% with a 4 wt.% BN inclusion. Aluminum nitride is a highly promising material that has attracted considerable attention for a wide range of applications, including for piezoelectric devices [20,21], microelectromechanical systems (MEMS) [22,23], the fabrication of Li-ion batteries with improved performance [24], supercapacitors [25], nano-electronics [26], nano-photonics [27], and quantum emitters [28].

An AlN is a wide-bandgap semiconductor that has been incorporated into nitride heterostructures for the fabrication of p-channel field-effect transistors (FETs), opening a route for next-generation electronics and the potential for mm-wave communication [29]. Moreover, AlN-based alloys have received particular interest because of the ferroelectric properties they demonstrate [30,31] and their tribological behavior [32]. Compared to other nitride compounds, aluminum nitride films exhibit high intrinsic phonon thermal conductivity values in both cross-plane and in-plane directions, making them suitable as heat spreaders in electronics [33,34]. As a result, the inclusion of nitride fillers in thermally insulating polymers has been proven to be an effective approach to improve the thermal conductivity of the matrix material. By adding BN platelets and AlN particles in polytetrafluoroethylene (PTFE), the cross-plane thermal conductivity of the composite was increased 3.8 times compared to that of neat PTFE [35]. The addition of modified AlN in the polyetherimide (PEI) matrix improved the electrical properties, thermal stability, and tensile strength of composites with the increase of AlN content, while a remarkable rise in the thermal conductivity was observed for the 57.4 vol.% PEI/AlN composite, being almost three times higher compared with the pure polymer [36]. Another report showed that by adding a hybrid filler consisting of AlN and MWCNTs in immiscible PC/Polyamide 66 (PA66) composites, an improvement in the thermal properties was achieved. Recently, Lin et al. reported an AM process of complex-shaped AlN-based components, evaluating their heat dissipation performance [37]. In general, very few studies have investigated the role of AlN nanoparticles in AM of nanocomposites using 3D printing, as well as their impact on the thermal and mechanical properties of the produced specimens.

MEX is the most widely used AM technology and has become the center of research in recent years due to the numerous benefits this technology can provide for large-scale manufacturing [38]. MEX requires materials with good thermal stability and mechanical properties. Investigations have focused on the optimization of 3D printing parameters and their impact on the mechanical and thermal response of the materials used for MEX [39,40,41]. Efforts have focused on optimizing the 3D printing conditions, in order to maximize their performance in AM [42], with respective research also conducted for the PC polymer studied herein [43]. The impacts of the fused filament fabrication (FFF) process, and more specifically the nozzle temperature and layer thickness parameters on the mechanical response of PC, were extensively studied in a recent study by this research team [44]. Other important parameters in the AM process, such as the heat transport performance and flow characteristics have been investigated with pulsating heat pipes fabricated by PC [45]. Computational investigations have also been employed, in order to develop models for the prediction of the mechanical behavior of materials used for 3D printing fused filament fabrication [46,47,48]. To enhance the mechanical performance of the polymers used in MEX 3D printing, various additives have been investigated and corresponding composites and nanocomposites have been developed [49,50,51,52,53,54,55,56]. PC polymers have been used as the matrix material for such nanocomposites, aiming to enhance the performance of parts fabricated with 3D printing [57]. Additionally, they have been used for the development of composites and blends, aiming to achieve improved mechanical performance [14,58,59,60,61,62].

Ceramics are materials with superior properties, therefore they are used in applications with extreme working conditions [63]. Therefore, ceramics have also been investigated as enhancement agents to improve the mechanical properties of MEX 3D printed parts. Specifically, for PC polymers, ceramics, such as silicon carbide [64], titanium nitride [65], and titanium carbide [16] have been used as additives in nanopowder form for the formation of corresponding nanocomposites. The research results showed remarkable improvements in the mechanical properties of the produced filaments, enhancing the ability of MEX 3D printing processes, thus showing the potential of such ceramic materials in nanopowder form. Additionally, the performance of the MEX 3D printed parts has been improved, in terms of their quality characteristics with post-processing procedures in hybrid material extrusion processes [66,67,68,69,70]. Attempts have also been made to improve the eco-friendliness of the 3D printing process [71,72], by optimizing the energy consumption [73,74], which as a result, affects the eco-friendliness of the produced parts.

In this study, and for the first time, the development of PC/AlN nanocomposites was carried out using the 3D printing MEX process. The impact of AlN nanoparticle content on the thermal and mechanical properties of the final nanocomposite filaments was thoroughly investigated. An evaluation of the feasibility of the thermomechanical MEX 3D printing process was carried out as well. Optimization of the mechanical reinforcement of the PC matrix was carried out through the MEX 3D printing process, which enabled the development of nanocomposite specimens with different AlN concentrations. The mechanical properties of the fabricated nanocomposites were tested following the American Society for Testing and Materials (ASTM) standards. Thermogravimetric analysis (TGA) and differential scanning calorimetry (DSC) were also carried out, to test the thermal stability of the developed nanocomposites, while Raman spectroscopy measurements revealed details about the composition and chemical bonds of the nanocomposite materials. Morphological characterization followed using atomic force microscopy (AFM) and scanning electron microscopy (SEM), in order to investigate the morphology and surface characteristics of the final specimens, from which important conclusions were extracted about the compatibility of the materials with the MEX 3D printing process. The optimum weight-to-weight percentage (wt.%) of AlN concentration was achieved for 2 wt.%, which presented the best mechanical behavior among all tested specimens, with notable enhancements in the tensile and toughness tests compared to the pure PC. AlN addition in the PC matrix induced a selective reinforcement of the mechanical properties, while all developed nanocomposites were compatible with the overall MEX process, without affecting their thermal stability and chemical composition. Therefore, it was concluded that the development of PC/AlN nanocomposites was successfully achieved and can open a new route for the expansion of 3D printing AM technology.

## 2. Materials and Methods

**Figure 1:**
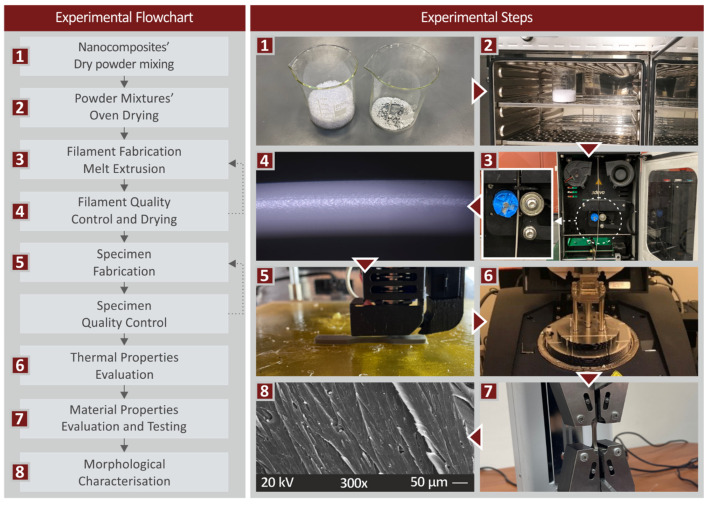
Steps of the experimental process.

Figure 1 illustrates the workflow of the experimental process.

### 2.1. Materials

The material extrusion process was used for the fabrication of nanocomposites. The matrix material (PC polymer) was EMERGE 8430–15, in the form of pellets with standard characteristics: a max strain of 110%, a tensile strength of 70.0 MPa, and a density of 1.20 g/cm^3^. It was purchased from Styron Europe GmbH (Trinseo Europe GmbH, Horgen, Switzerland). The additive was AlN in nanopowder form, procured from Nanographi (Nanografi Inc., Ankara, Turkey), with a purity of 99.95%, density of 0.05 g/cm^3^, size of 60–70 nm, and hexagonal shape for the nanoparticles. Since the size of the particles according to the manufacturer was 60–70 nm, the D90 classification for the particle size was 70 nm.

### 2.2. Preparation of Nanocomposites

Initially, a drying process for 14 h was implemented for the raw materials using a dryer at 60 °C, for the removal of possible moisture. Four different types of raw material powder mixture were prepared, one for each different weight-to-weight (wt.%) AlN content: 1.0, 2.0, 3.0, and 6.0 wt.%. The mixtures of AlN nanopowder with the powder of PC matrix were carried out using a high-power blender inside a glove box at a room temperature of 23 °C and 4000 rpm for 30 min. A drying process followed for the appropriate powder mixtures, to achieve an initial dispersion of AlN nanopowder in the polymer matrix, and subsequently they were transferred into a single screw Noztek extruder (Noztek, Shoreham-by-Sea, UK). The produced filament was converted to pellets using a 3devo shredder (3devo, Utrecht, The Netherlands). Subsequently, a 3devo Composer (3devo, Utrecht, The Netherlands) equipped with a special screw design for mixing materials and additives was used to fabricate the filaments with a diameter of 1.75 mm, appropriate for 3D MEX printing. The temperature setting for the first three heating zones was 200 °C, while the fourth heating zone operated at 240 °C. The screw was operated at 4.8 rpm. The two extrusion steps were implemented, in order to achieve an effective dispersion of the filler in the PC matrix. It should be noted that no other additives, such as compatibilizers or others, were used for the nanocomposite preparation, in order to study the clear effect of the additive in the matrix material. Filament from the pure polymer was also fabricated following the same extrusion steps, in order to make a comparative evaluation of the mechanical and thermal performance of the nanocomposites.

### 2.3. Specimens Fabrication

**Figure 2:**
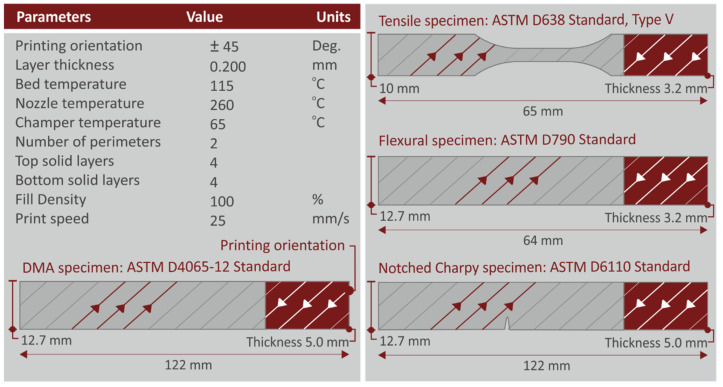
The 3D printing parameters for the manufacturing of specimens. The specified dimensions of the fabricated specimens for each mechanical test are presented on the right side.

Filaments of all PC/AlN nanocomposites and that of the pure polymer developed by the previous process were used for the manufacturing of specimens, in order to assess their mechanical properties. For this purpose, a Funmat HT 3D printer (Intamsys, Shanghai, China) was used. The parameters set-up for the 3D printing process are presented in Figure 2 and were determined experimentally, according to the compatibility of the materials. Intamsuite software was used for the preparation of G-codes. Figure 2 also shows the dimensions of the specimens for each mechanical test conducted under international standards (ASTM D638-02a for the tensile tests, ASTM D790-10 for the flexural tests and ASTM D6110-02 for the Charpy notched impact tests).

### 2.4. Thermal Analysis and Raman Spectroscopy

TGA and DSC measurements were carried out to determine the thermal stability of the materials used in the study, especially at the temperatures where the extrusion process took place. TGA measurements were carried out using a Perkin Elmer Diamond (Perkin Elmer, Waltham, MA, USA) instrument under a nitrogen atmosphere, in a temperature range of 40 ≤ T/°C ≤ 550 with a step of 10 °C/min, while a DSC 25 from TA Instruments (TA Instruments, New Castle, DE, USA) was used for DSC in the range of 25 ≤ T/°C ≤ 225 with a step of 15 °C/min. A Raman spectrometer (HORIBA Scientific, Kyoto, Japan) was also used for the characterization of the investigated nanocomposites, as well as the pure PC polymer. The instrument was equipped with a solid-state laser with a central wavelength of 532 nm, a maximum output power of 90 W, and an objective lens operating with 0.5 numerical aperture and 50× magnification. For all measurements, a working distance of 10.6 mm was set, while the laser spot had dimensions of about 2 μm axially and 1.7 μm laterally. An ND filter was applied to control the laser light, allowing 5% to pass through and corresponding to a power of 2 mW. A spectrometer with a ~2 cm^−1^ resolution operated in a spectral range from 300 to 3100 cm^−1^ using two acquisitions for each point, with a time of 10 s per acquisition and 5 accumulations for each measurement. The processing of Raman data was carried out using LabSpec 6 from HORIBA Scientific (HORIBA Scientific, Kyoto, Japan). A polynomial function was used for the subtraction of the background and with a unit vector used being for the data normalization of all samples.

### 2.5. Filament Evaluation

All produced filaments were evaluated by conducting tensile tests and characterizing their surface morphology. Real-time measurements of the filament diameter were carried out during the experimental process using the closed-loop measuring instrument of the extruder. The results were cross-checked with measurements using an electronic caliper. An Imada MX2 apparatus (Northbrook, IL, USA) was used for the tensile strength measurements. The tensile tests for the filament were not conducted following a standard. The standard grips for the dogbone samples were also used for the fixture of the filament in the machine. The test speed was set to 10 mm/min, to be the same as in the dogbone specimens experiments that followed. In addition, for each nano-compound, five filament samples were tested. The surface characteristics of the filaments were investigated through AFM, using a Solver P47H Pro instrument (MicroscopeSolver P47H Pro, Moscow, Russia) with a resonant frequency of 300 kHz.

### 2.6. Mechanical Tests

Table 1 presents all mechanical tests (and their settings), which were conducted in order to evaluate the mechanical performance of the nanocomposites in comparison with the pure PC. Five samples were tested in each test, and all tests were conducted under room temperature conditions (23 °C and humidity of 55%).

### 2.7. Morphology Characterization of Specimens

The morphological characteristics of the fabricated specimens were studied using scanning electron microscopy (SEM) (JEOL JSM 6362LV, Peabody, MA, USA, 20 kV). All samples were gold-sputtered under a high vacuum, while energy dispersive spectroscopy (EDX) analysis was also carried out for uncoated samples.

## 3. Results

### 3.1. Thermal Analysis and Raman Spectroscopy

**Figure 3:**
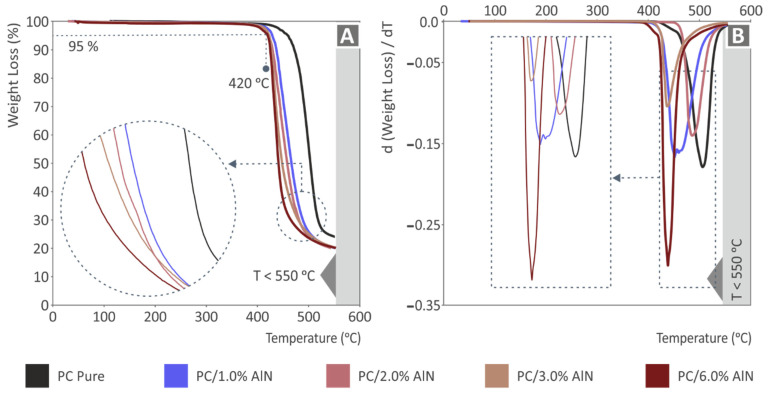
TGA measurements for all PC/AlN nanocomposites and pure PC: (**A**) weight loss and (**B**) its derivative dw/dT as a function of temperature.

Figure 3A shows TGA graphs with the weight loss of the nanocomposites and the pure PC as a function of temperature. As can be observed, the addition of AlN in the PC matrix had a clear impact on the thermal stability of the nanocomposite materials. While the weight loss of pure material started at about 465 °C, the weight loss of nanocomposites was reduced to 95% at lower temperatures. More importantly, the starting temperature of material degradation followed a gradual decrease with the increase of AlN content. As a result, the intense weight loss of PC/AlN 6.0 wt.% started at ca. 420 °C. However, this temperature was still much higher than the temperatures employed in the material extrusion and, consequently, it was verified that the thermal behavior was not affected by the extrusion process. The temperature where the maximum weight loss rate occurred also presented a decreasing exponential trend as a function of AlN content (Figure 3B). Therefore, the maximum weight loss rate of pure material was observed at about 500 °C, while for the nanocomposite of 6 wt.% AlN, the corresponding temperature was ca. 440 °C. It is also notable that PC/AlN 6.0 wt.% exhibited a relatively higher value of maximum loss rate (*ca*. -0.3) compared to those of the lower AlN-content nanocomposites and pure polymer.

**Figure 4:**
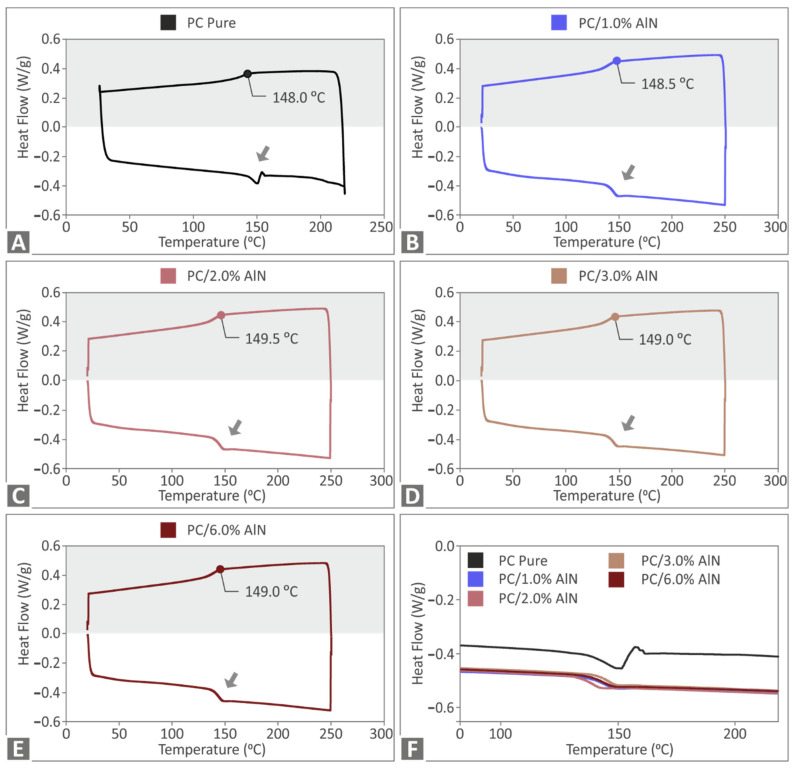
Heat flow curves (exothermic and endothermic) as a function of temperature for (**A**) pure PC, (**B**) PC/AlN 1 wt.%, (**C**) PC/AlN 2 wt.%, (**D**) PC/AlN 3 wt.%, (**E**) PC/AlN 6 wt.%, and (**F**) comparison of exothermic curves. Arrows indicate the phase transition of the material.

**Figure 5:**
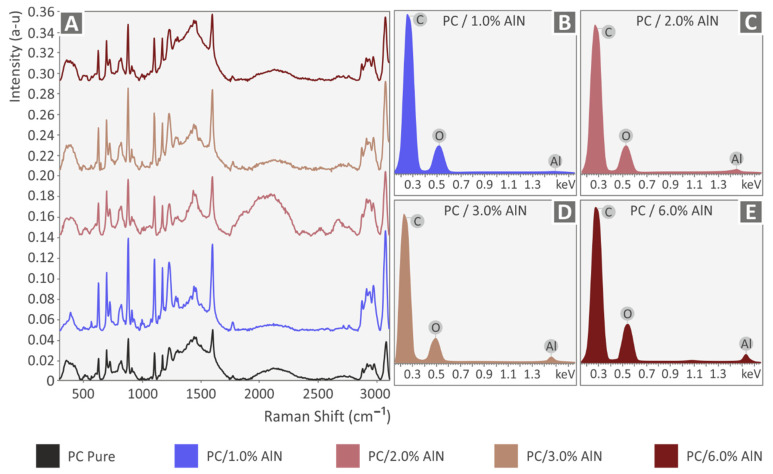
(**A**) Raman spectra for all materials, EDS analysis for nanocomposites with (**B**) 1 wt.% AlN, (**C**) 2 wt.% AlN, (**D**) 3 wt.% AlN, and (**E**) 6 wt.% AlN.

In Figure 4, the corresponding DSC data of all samples, including the pure polymer, have a similar response, without showing a great effect of filler addition on the characteristic temperatures of the materials. However, it must be noted that AlN addition enhanced the energy absorbed in both the endothermic and exothermic stages, with minor changes among PC/AlN nanocomposites. The Raman spectroscopy results for PC/AlN nanocomposites and pure PC are presented in Figure 5A. All peaks identified in the range between 573 cm^−1^ and 3073 cm^−1^ corresponded to assignments of the Raman modes of the pure polymer, according to previously published studies [65,75,76,77].

### 3.2. Filament Characterization

**Figure 6:**
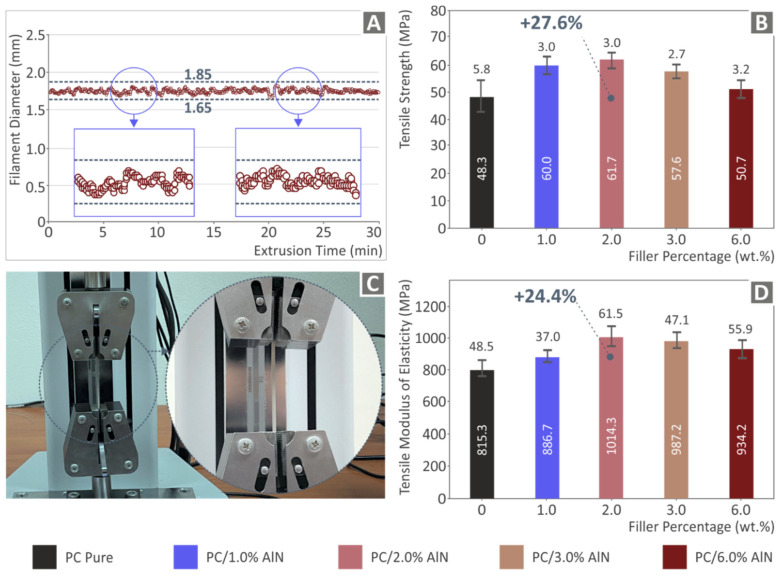
Characterization of all filaments tested: (**A**) real-time filament diameter measurements during the fabrication process, (**B**) tensile strength results (average values and deviation), (**C**) equipment for the conduction of tensile tests, and (**D**) tensile modulus of elasticity (average values and deviation) of the five tested filaments.

For the extrusion of the filament, a 3devo composer extruder (3devo, Utrecht, The Netherlands) was used. This extruder features a real-time filament diameter system, which operates in a closed loop. It measures the produced filament diameter and automatically adjusts the extrusion settings within an acceptable limit, in order to produce as accurate filament in diameter as possible. Real-time filament diameter measurements were conducted using the extruder integrated system, as presented in Figure 6A. Figure 6A shows a randomly selected segment of these measurements, in which the deviation in the produced diameter is shown. The deviation shown was the highest measured during the production of the filament, and this limit was not exceeded in the work. It should be noted that when defining the 3D printing parameters in the 3D printer software, the filament diameter is defined by the user, and this is considered when 3D printing the parts, as it affects the nozzle trajectory. Overall, the diameter values of the fabricated filaments exhibit good consistency, with a slight deviation, appropriate for MEX 3D printing.

Figure 6C illustrates the experimental setup used for the tensile tests. As can be observed in Figure 6B, the tensile strength of PC/AlN nanocomposites presented higher values than those of the pure material. Therefore, it was concluded that the AlN addition enhanced the tensile strength of the polymer matrix. The nanocomposite with 2 wt.% AlN exhibited a maximum value of ca. 62 MPa, which corresponds to an increase of 27.6% compared to that of the pure material. Moreover, the addition of AlN resulted in an improvement of stiffness, as observed in Figure 6D. The highest increase in stiffness was also presented by the 2 wt.% AlN content, with a percentage increase of 24.4%. Above the percentage of 2 wt.%, both the tensile strength and the stiffness were gradually reduced with the increase of AlN content. This means that this point presented a threshold and the mechanical properties of the filament had reached their optimum values.

**Figure 7:**
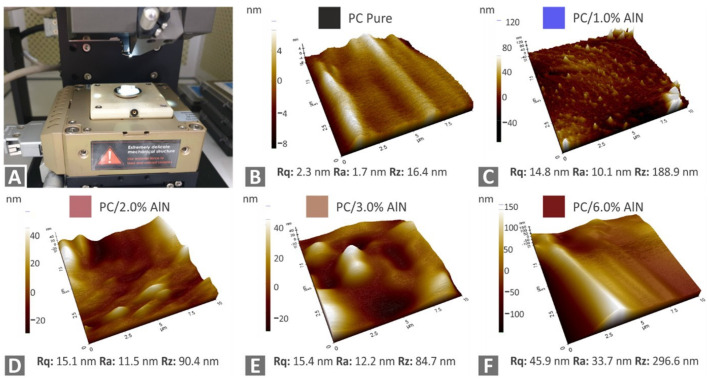
AFM measurements for the study of surface characteristics of tested filaments: (**A**) AFM instrument illustration; images for (**B**) pure PC (**C**) PC/1 wt.% AlN (**D**) PC/2 wt.% AlN, (**E**) PC/3 wt.% AlN, and (**F**) PC/6 wt.% AlN.

Subsequently, a morphological characterization was conducted on the side surface of the tested filaments using AFM (Figure 7). The pictures show that the filament surface roughness of all nanocomposites was higher compared to the pure polymer by values of more than one order of magnitude. Moreover, it was observed that the surface roughness of the nanocomposites presented a gradually increasing trend as the AlN content increased in the polymer matrix, indicating that AlN nano-powder greatly affected the surface morphology of the produced filaments.

### 3.3. Mechanical Characterization of the Fabricated Specimens

**Figure 8:**
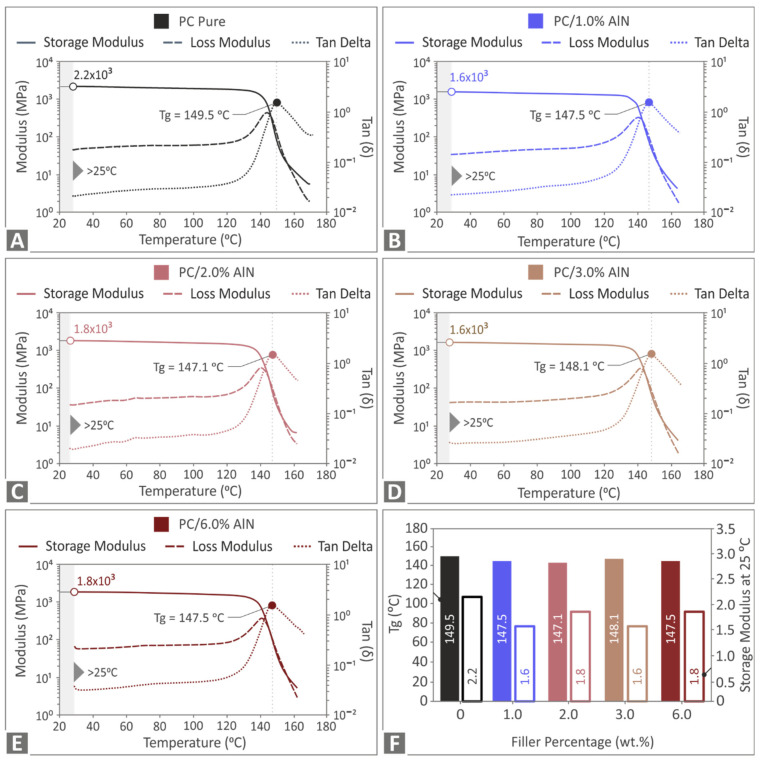
DMA results of storage and loss modulus, tan(delta)) for (**A**) pure PC, (**B**) PC/AlN 1 wt.%, (**C**) PC/AlN 2 wt.%, (**D**) PC/AlN 3 wt.%, (**E**) PC/AlN 6 wt.%, and (**F**) the calculated Tg (solid color bars) and storage modulus values (outline bars) at room temperature for all tested specimens.

A DMA test was conducted for the pure polymer and the nanocomposites, to study the viscoelastic behavior of the materials in this work. Figure 8 presents the thermograms of all tested samples. As can be seen, there were no notable differences in the curves, with the storage modulus and loss modulus following typical trends in the glassy phase. In the temperature region, the storage modulus was reduced sharply, and an increase in the loss modulus was simultaneously observed, which was indicative of the glass transition from the glassy phase to the rubbery state. However, it must be noted that a slight decrease was observed in the storage modulus and the glass transition temperature of the nanocomposites compared to that of the pure PC. A drop in Tg by 2.4 °C was presented by the PC/AlN 2 wt.%, reaching a value of 147.1 °C.

Therefore, the results showed that AlN loading had a moderate impact on the viscoelastic behavior of the PC, without a clear trend between Tg and the AlN content. As was suggested in a previous study [19], the reduction in Tg of PC with boron nitride addition was attributed to the higher free volume that existed among polymer chains and enabled a higher segment mobility of the polymer molecules at the region close to the air–polymer interfaces. Possibly there also was no positive interaction between the matrix and the nanoparticles for the case of PC/AlN, consequently enabling the segment mobility of PC molecules.

**Figure 9:**
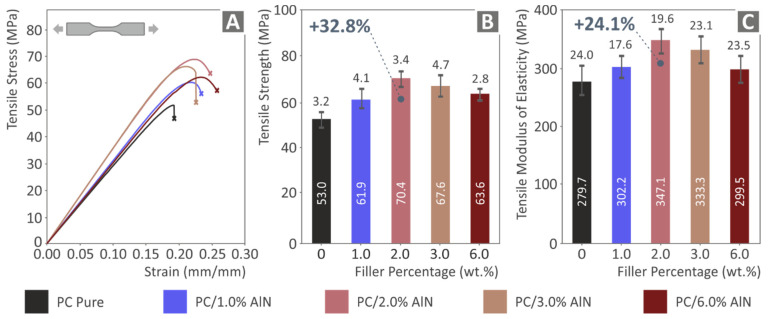
Tensile tests results of the 3D printed samples: (**A**) tensile stress graph as a function of strain, (**B**) tensile strength (average values and deviation), and (**C**) tensile modulus of elasticity (average values and deviation) of the five tested specimens.

The results from the tensile experiments are presented in Figure 9. It was observed that, overall, the loading of AlN nanoparticles enhanced the tensile strength, since all nanocomposites presented increased values compared to that of the pure polymer. The nanocomposite with 2 wt.% AlN exhibited the highest increase, with a percentage of 32.8%, while above this point a slight decrease was observed, indicating that the tensile strength had reached a threshold as a function of AlN wt.%. Moreover, the addition of AlN nanoparticles had a positive effect on the tensile modulus of elasticity, making the nanocomposites stiffer in comparison with the pure polymer. The concentration of 2 wt.% also demonstrated the maximum value of tensile modulus of elasticity, which was 24.1% higher than the pure PC. The addition of AlN nanoparticles into the PC matrix increased the developed strain of the samples compared to the pure PC material, as shown in Figure 9A. The highest increase in the strain was presented for the highest loading of nanocomposite (6 wt.%), but the overall the effect of AlN addition in the strain was rather similar between the nano-compounds.

**Figure 10:**
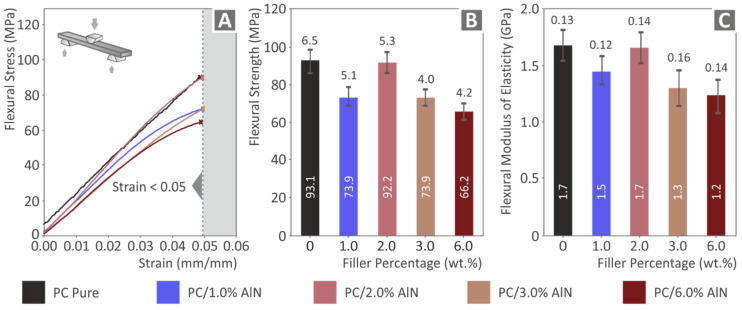
Flexural tests results on the 3D printed samples: (**A**) flexural stress graph as a function of strain (termination at 5% strain), (**B**) flexural strength results (average value and deviation), and (**C**) flexural modulus of elasticity results (average value and deviation) of the five tested specimens.

In contrast with the tensile tests, the flexural test results presented in Figure 10 show that AlN loading weakened the flexural mechanical response of the nanocomposites, with the lowest values being demonstrated by the nanocomposite with the highest AlN concentration (6 wt.%). However, the material with 2 wt.% presented almost the same values for both flexural strength and modulus of elasticity as those of the pure PC, exhibiting resistance to the reduction trend. Regarding the flexural tests, no conclusions can be drawn for the effect of the AlN addition in the strain developed on the samples during the tests, as the experiments were terminated at a 5% strain, following the instructions of the ASTM D790 standard.

**Figure 11:**
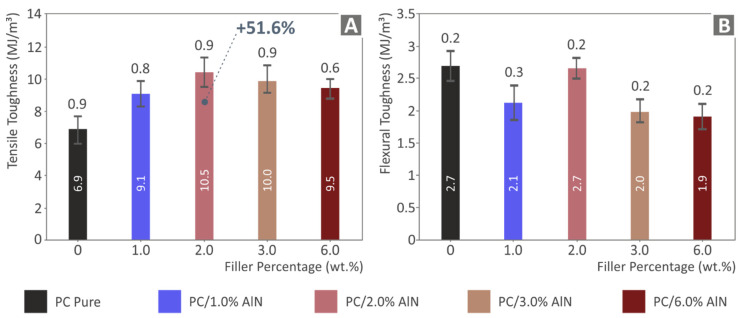
(**A**) Tensile toughness results (average value and deviation), (**B**) flexural toughness results (average value and deviation) of the five tested specimens.

Tensile and flexural toughness values were extracted from the integrals of the corresponding stress versus strain graphs and are indicative of the energy absorbed by the tested samples. Figure 11 presents the average values for all tested specimens, as well as their deviations. It is obvious that they present a similar trend to the corresponding strength values. The tensile toughness of the 2 wt.% AlN nanocomposite showed an increase of 51.6% over that of the pure polymer, while its flexural toughness reached the same value as PC. The strain developed on the samples during the tests also contributed to the toughness values. Therefore, the increased calculated toughness, at least in the tensile tests (since the flexural tests were terminated at the same strain value of 5%, as mentioned above), could be attributed not only to the increased strength but also to the increased strain of the nanocomposites in the tests, compared to the pure PC polymer.

**Figure 12:**
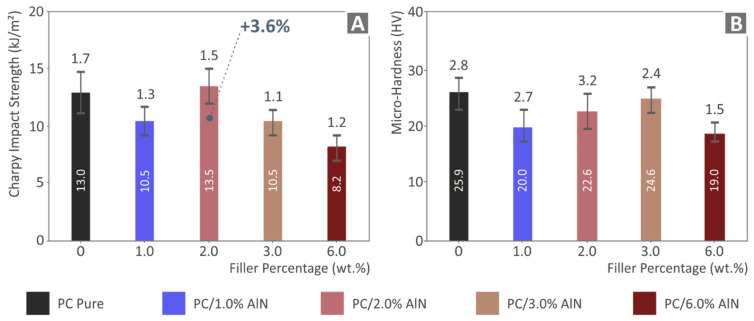
(**A**) Impact strength results (average value and deviation) (**B**) Vickers microhardness results (average value and deviation) of the five tested specimens.

On the other hand, as can be observed in Figure 12, the impact test results had a different trend compared to the previous mechanical tests. Apart from the slight increase of 3.6% in the impact strength of 2 wt.% content-nanocomposite in comparison with the unloaded PC, all the other nanocomposites presented lower values. The Vickers micro-hardness results also presented the negative effect of AlN inclusion in the PC matrix, since all AlN-nanocomposites exhibited lower values than that of the pure polymer. It must be noted that the material with the highest AlN concentration of 6 wt.% had the lowest levels for both impact strength and micro-hardness.

### 3.4. Morphology Characterization of Fabricated Specimens

**Figure 13:**
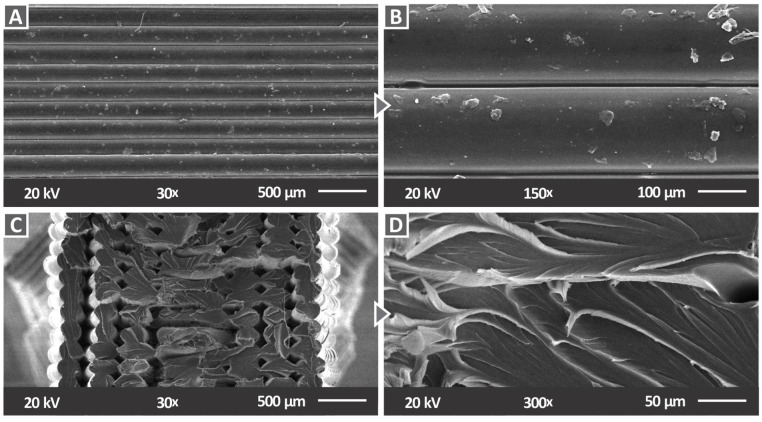
SEM images for pure PC: (**A**) side surface with 30× magnification, (**B**) side surface with 150× magnification, (**C**) fracture surface with 30× magnification, and (**D**) fracture surface with 300× magnification.

Figure 13A, B illustrate images of the side surface of the pure polymer captured by SEM at two different zoom levels. As can be seen, no defects or voids are observed at the side surface, which exhibits a perfect layer interfusion, indicating a high-quality level in the 3D printing process. These results confirmed that the appropriate parameters were set for the process of 3D printing, being compatible with the characteristics of the PC polymer. Figure 13C, D present the fracture surface of the pure PC at a magnification of 30× and 300×, respectively. On the right side of Figure 13C, there are areas where the filament strands do not show any deformation, which is indicative of a brittle failure, while the left side presents a visible deformation of the filament strands, indicative of a more ductile failure.

**Figure 14:**
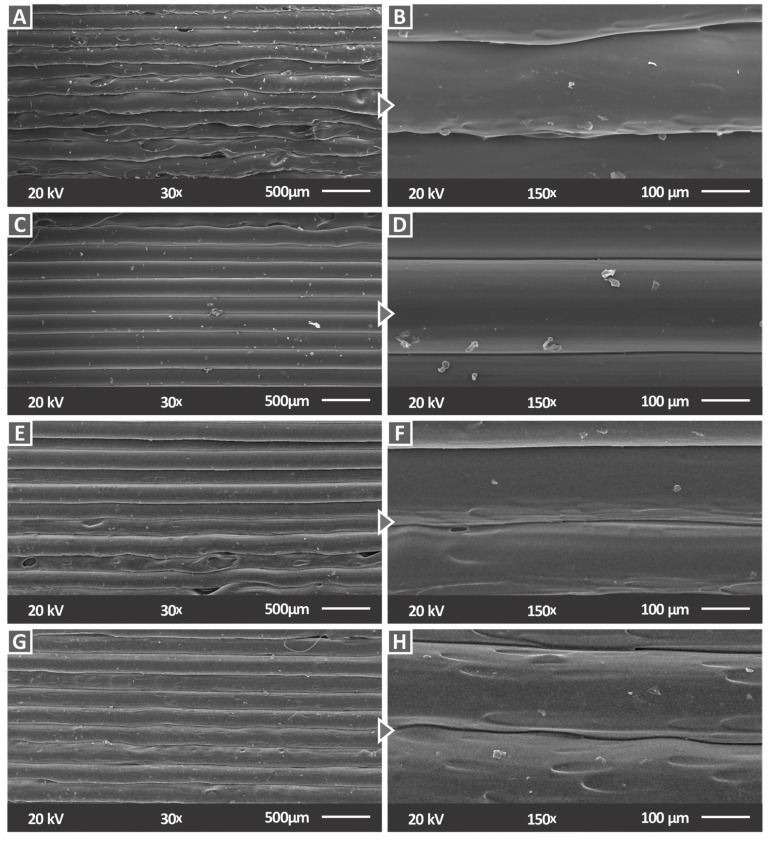
SEM images of the side surface for (**A**) PC/AlN 1 wt.% with 30× magnification, (**B**) PC/AlN 1 wt.% with 150× magnification, (**C**) PC/AlN 2 wt.% with 30× magnification, (**D**) PC/AlN 2 wt.% with 150× magnification (**E**) PC/AlN 3 wt.% with 30× magnification, (**F**) PC/AlN 3 wt.% with 150× magnification, (**G**) PC/AlN 6 wt.% with 30× magnification, and (**H**) PC/AlN 6 wt.% with 150× magnification.

Figure 14 illustrates SEM side surface images of the investigated PC/AlN nanocomposites at 30× and 150× magnification. Compared to the results of the pure PC, the side surface of all nanocomposites exhibited an excellent layer interfusion, indicating that the 3D printing quality remained at the same high level with the inclusion of AlN nanoparticles.

**Figure 15:**
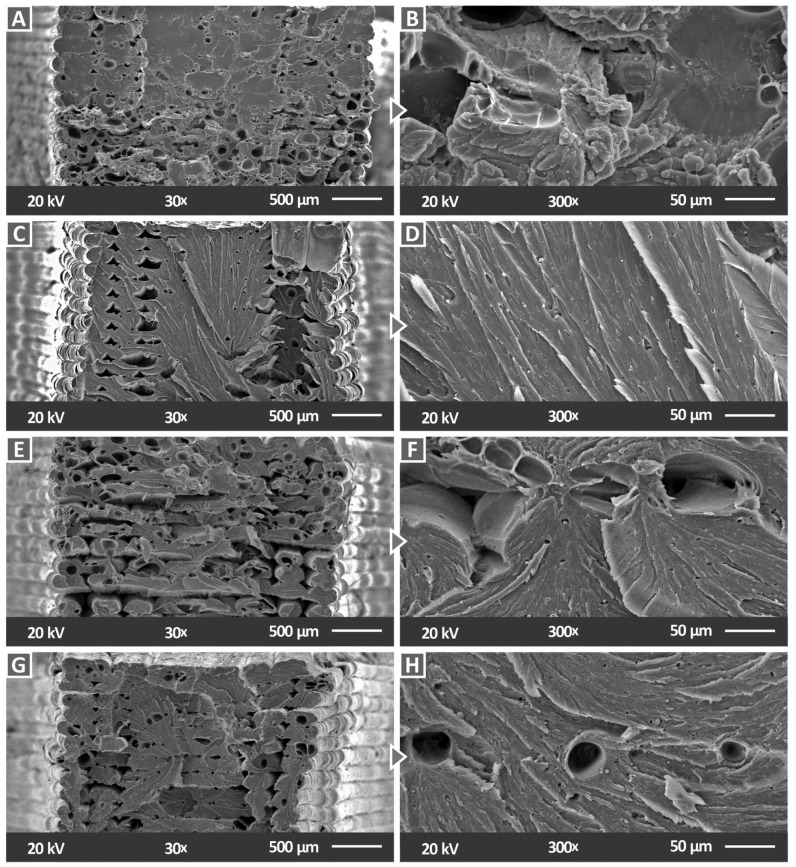
SEM images of fracture surface for (**A**) PC/AlN 1 wt.% with 30× magnification, (**B**) PC/AlN 1 wt.% with 300× magnification, (**C**) PC/AlN 2 wt.% with 30× magnification, (**D**) PC/AlN 2 wt.% with 300× magnification, (**E**) PC/AlN 3 wt.% with 30× magnification, (**F**) PC/AlN 3 wt.% with 300× magnification, (**G**) PC/AlN 6 wt.% with 30× magnification, and (**H**) PC/AlN 6 wt.% with 300× magnification.

SEM images of the fracture surfaces with high and low magnification rates were also taken for all nanocomposite samples, as illustrated in Figure 15. Such images are standard fractography images for the evaluation of the fracture mechanism in samples and for the correlation with mechanical tests. Up to the 2 wt.% filler loading, the nanocomposites had minimum pores, and this agreed with the high mechanical test results (the highest values were reported at the 2 wt.% loading). For higher filler loadings, the number of pores in the fracture increased, having a negative effect on the mechanical strength of the samples. The structure shown in the fracture surfaces (pores and voids) was the expected one for MEX 3D printed parts, and this affected the mechanical response of the parts [78,79].

For the 1 wt.% AlN inclusion, minimum deformation of the filament strands was observed (Figure 15A,B). A mixed fracture surface was observed for 2 wt.% AlN. The strands on the two sides exhibited minimal deformation, while a brittle behavior was observed in the middle of the specimen. The fracture surface image of the material with 3 wt.% AlN loading shows a ductile response, with a visible deformation in the filament strands. A mixed fracture surface can also be observed for the 6 wt.% AlN-nanocomposite, with certain strands exhibiting minimum deformation, mostly on the sides of the specimen, while the middle area was characterized by a brittle behavior.

**Figure 16:**
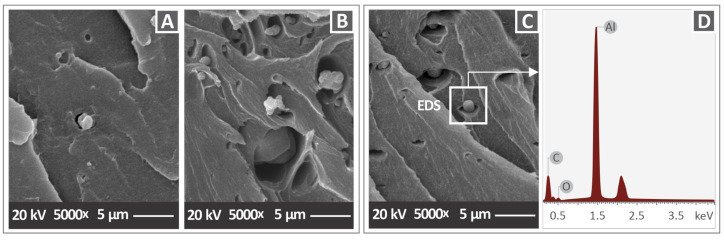
SEM images of fracture surface with a magnification of 5000× magnification of (**A**) PC/AlN 1 wt.% (**B**) PC/AlN 3 wt.%, (**C**) PC/AlN 6 wt.%, and (**D**) EDS analysis of PC/AlN 6 wt.% nanocomposite, obtained on the agglomeration, as illustrated in (**C**).

SEM images with a magnification of 5000× are presented in Figure 16 for the nanocomposites with 1 wt.%, 6 wt.%, and 3 wt.% AlN concentration. As can be observed, the distribution of agglomerations gradually followed the increase of AlN concentration in the PC matrix, and as a result most agglomerations corresponded to the nanocomposite with the highest AlN concentration of 6 wt.%. Figure 16C shows the EDS analysis of an agglomeration region that was captured for the sample with 3 wt.% filler loading. As expected, a high peak is observed close to 1.5 keV, which corresponds to Al, implying a high content of this element and confirming the existence of AlN nanoparticles.

## 4. Discussion

**Figure 17:**
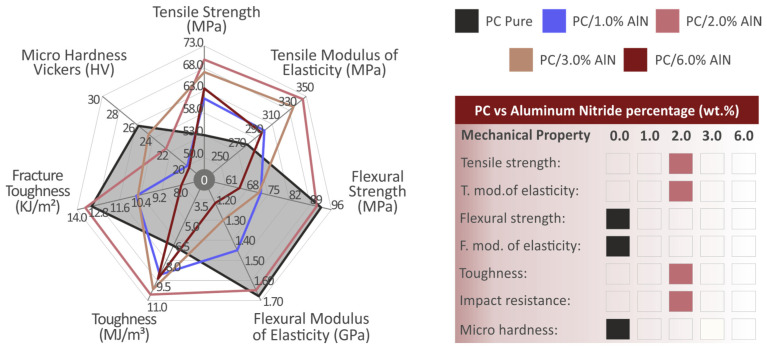
Summary of mechanical test results, depicted in a spider diagram. The shaded area corresponds to the pure PC. The right table presents the materials with the highest performance for each mechanical test.

According to the mechanical test results summarized in Figure 17, among the nanocomposites, the one with 2 wt.% filler exhibited the optimum mechanical response. For this concentration, most of the mechanical properties were improved, while the others remained almost at the same level as those of the pure polymer. The highest improvements were observed in the tensile and toughness tests. It should be noted that this work aimed to reinforce the PC matrix material. A 100% infill material was selected, since this provided the maximum mechanical strength results [80,81], and therefore the maximum effect on the PC matrix. Since MEX 3D printed parts have an inferior mechanical strength to the corresponding injection molded parts [82,83,84], when the aim is to produce functional parts with the MEX 3D printing process, the maximum strength needs to be achieved in the 3D printed parts. Various works have studied the effect of 3D printing parameters on the mechanical strength of 3D printed parts, with the aim of optimizing the mechanical strength of the 3D printed parts [85,86,87].

It must be noted that, although there was an overall reduction of the flexural mechanical properties with the inclusion of filler, the 2 wt.% AlN-nanocomposite resisted this decrease and exhibited values similar to the pure PC, resulting in an overall improvement of the mechanical properties. The Vickers micro-hardness was the only property that showed a reduction for all nanocomposites. The micro-hardness on the surface of samples was studied in this work, as it is an indication of the wear resistance of the samples [88,89,90]. In addition, from the test results, it was shown that the nanocomposites with concentrations above 2 wt.% presented a gradual decrease compared with the upper threshold of 2 wt.%. This implies that the optimization of mechanical properties had been achieved through the control of AlN concentration. The fact that the nanocomposites with AlN concentrations above 2 wt.% exhibit weakened flexural properties can be explained by the possible saturation of the AlN additive in the PC matrix. It is probable that the 2 wt.% loading was close to the percolation threshold for the AlN additive in the PC matrix. However, the exact percolation was not identified in this work, as it was not within the purposes of the work. This was evaluated through the decrease in the mechanical test results. For the nanocomposite with 6 wt.% AlN content, the mechanical performance could be also attributed to the higher distribution of agglomerations, which was revealed in the high-magnification SEM images.

Overall, following the two-step extrusion process (two successive extrusion processes, with the second one on an extruder specially designed for material mixing), a good dispersion of the AlN additive in the PC matrix was achieved. This was verified by the mechanical test results, in which the calculated deviation in all the conducted experiments was within acceptable limits, showing that the nanocomposites had the same composition and structure in all cases tested. Additionally, the morphological examination of the specimens with SEM agglomerations was located only in the nanocomposite with the highest AlN concentration studied in the work. It should be noted that the distribution of the nanoparticles was investigated after the 3D printing process with the specimens. The distribution of the filler in the matrix is expected to be the same before (in filament form) and after the 3D printing process. When the filament manufacturing process is completed, the mixing procedures are completed as well. The 3D printing process is not expected to change the distribution of the filler in the matrix, as it is a melt-and-flow process.

The elemental analysis of EDS confirmed the composition of nanoparticle agglomerations. Although there was a small reduction in the thermal stability of the polymer with the addition of AlN nanoparticles, it was confirmed that the temperatures set for the 3D printing were too low to cause any degradation effect in the investigated materials. Raman spectra did not show any changes in the graphs of the nanocomposites in comparison with that of the pure polymer, which means that all Raman modes are attributable to the PC polymer. The concentration of the AlN additive was quite low, up to 6 wt.%, and possibly for this reason, new peaks attributed to AlN were not detected in the data of the nanocomposites. In addition, no shift or change in the intensity was observed in the Raman modes of the PC polymer with the increase of the AlN concentration, implying that AlN nanoparticles did not affect the chemical bonds of the PC polymer, and consequently, it was confirmed that there was no chemical interaction between the two phases, as has also been suggested in the case of PC/BN nanocomposites [19].

The inclusion of AlN nanoparticles in the PC polymer matrix provided effective enhancements of the tensile strength and toughness of the materials, opening a new direction for the development of 3D printing and demonstrating a new application process. Comparing the test results of this work with those of a previous study of this research team on PC/TiN nanocomposites, a different mechanical response of the two systems is observed. The main difference is that for the PC/TiN nanocomposites there was an overall enhancement of mechanical properties, including the flexural and micro-hardness test results. In addition, the optimum value of TiN concentration was at 3 wt.%, for which the best mechanical properties results were achieved. This amount is a little higher than that obtained in this work. Comparing the results of AlN/PC in this study with those of f-BNNT/PC nanocomposites developed by melt-mixing-extrusion and compression molding process, it can be concluded that AlN nanoparticles induce a stronger enhancement of tensile properties than BN nanotubes [19]. This can be explained by the fact that the dispersion of filler in the PC matrix was lower in the case of BN nanotubes, where large aggregates and impurities were revealed in the SEM images. In this study, agglomerations of AlN nanoparticles were also observed, but to a lesser extent and mostly for the high AlN concentration sample with 6 wt.%.

On the contrary, TiN nanoparticles presented a quite effective dispersion in the PC matrix, without the presence of agglomerations [65]. For this reason, it is likely that PC/TiN nanocomposites would exhibit an overall improvement of mechanical properties, including the micro-hardness and flexural strength, compared to the results observed for the PC/AlN system. Therefore, it is concluded here that the efficient dispersion of nano-additives in the polymer matrix seemed to strongly affect the mechanical performance of the developed nanocomposites. This is probably closely related to the interfacial interaction between the additive and the matrix material. It is worth noting that in both cases, with BN and AlN nanocomposites, the interaction of nanofillers with the PC matrix seemed to be weak or negligible. This may also explain the fact that for both systems a reduction in T_g_ was observed with the inclusion of nitride additives, since the absence of positive interactions between the matrix and the nanofillers enabled the segment mobility of PC molecules and the transition from the glassy phase to the rubbery state at slightly lower temperatures. Here, the Raman spectra confirmed no chemical interaction between the AlN nanoparticles and PC polymer, while in the case of TiN addition, an increase in the intensity of high-dissociation energy C-H bonds was observed for the PC/TiN nanocomposites, which may also have strongly affected the overall mechanical reinforcement of the materials.

The effect of the addition of ceramic-type nanoparticles in the PC matrix material for MEX 3D printing applications has also been presented in the literature for other ceramics [16,64]. These works aimed to investigate the reinforcement effect with the addition of each filler. Similar filler loadings were investigated, and nanocomposites were prepared using a similar process. When comparing the results to the current work, similar patterns can be observed. In both works, the 2 wt.% filler loading nanocomposite exhibited the highest reinforcement. The thermal stability and the processability of the PC material were not affected by the loadings studied. The reinforcement effect was higher with the titanium carbide additive (41.9% in the tensile strength) [16] and lower with silicon carbide (19.5% in the tensile strength) [64], compared to the results of the current work. Such differences can be attributed to the different additive properties and the different interactions between the additive and the PC polymer in each case.

## 5. Conclusions

In this study, the addition of AlN nanoparticles in the PC matrix and its effect on the mechanical and thermal performance of the developed nanocomposites were investigated. The nanocomposites were fabricated using MEX 3D printing with various amounts of AlN, up to 6 wt.%, and an intensive morphological and mechanical characterization was conducted, to evaluate the 3D printing process, the setting parameters, and the mechanical behavior of the manufactured specimens. The inclusion of AlN induced a selective reinforcement of the mechanical properties, with the best performance achieved for the nanocomposite with 2 wt.% AlN content. Specifically, an improvement of the tensile strength and tensile modulus of elasticity was observed, with percentages of 32.8% and 24.1%, respectively. In addition, a high increase of 51.6% was noted for the tensile toughness, while the impact strength of 2 wt.% AlN also showed a slight enhancement, with a percentage of 3.6%. On the other hand, the microhardness and flexural tests showed a reduction of nanocomposite properties compared to the pure polymer. However, the PC/2 wt.% AlN nanocomposite presented resistance to this decreasing trend, exhibiting flexural strength and flexural modulus of elasticity values close to the levels of pure PC. This may be explained by the fact that, for low concentrations, the AlN nanoparticles still presented a sufficient dispersion in the matrix. On the contrary, for higher concentrations, such as 3 and 6 wt.%, high-magnification SEM images revealed the formation of nanoparticle agglomerations in the PC matrix.

Thermal analysis confirmed that the inclusion of AlN nanoparticles in the PC matrix was compatible with the MEX 3D process. Although the addition of AlN moderately affected the thermal stability of the polymer, reducing the starting temperature of weight loss to 420 °C, it was verified that no degradation effect was induced during the 3D printing process, since the maximum setting temperature (270 °C) was much lower than that value. DMA measurements showed a slight decrease in the glass transition temperature with the introduction of AlN nanoparticles. The nanocomposite with 2 wt.% AlN exhibited a Tg of 147.1 °C, which was 2.4 units lower than that of pure PC. This may be attributed to the fact that there was no chemical interaction between the two phases. This, together with a sufficient free space between PC molecules, allowed the increase of segment mobility in the vicinity of PC/AlN interfaces, resulting in a quicker transition from the glassy to a rubbery state. Raman spectroscopy measurements confirmed that there were no changes in the bonds of the PC molecules and no interactions between the two phases. Therefore, it is concluded that the addition of the small amount of 2 wt.% AlN in the PC polymer enhanced the mechanical performance of the specimen manufactured by MEX 3D printing, simultaneously inducing a small effect on the thermal behavior of the polymer matrix, to an extent that was not detrimental to the 3D printing process.

Finally, this study demonstrated the successful AM of PC/AlN nanocomposites through MEX 3D printing, which was compatible with the incorporated materials. In future work, further investigations should be conducted, for instance on the thermal transport properties (conductivity and diffusivity), in order to obtain a complete image of the impact of the AlN additive on the PC polymer. This will further enlighten the research on 3D printing AM, expand its applications, and contribute to the development of multifunctional nanocomposite materials.

## Figures and Tables

**Table 1:** Mechanical Characterization Tests.

**DMA**	
Test	Three-point bending
Temperature Range	30–200 °C
Temperature Rate	5 °C/min
Oscillation Magnitude	30 μm
Frequency	1 Hz
Preload	0.1 N
Standard	ASTM D4065—12
Device	DHR 20 of TA Instruments (TA Instruments, New Castle, DE, USA)
**Tensile**	
Sample	Type V with a thickness of 3.2 mm
Strain rate	10 mm/min
Standard	ASTM D638—02a
Device	Imada MX2 (Northbrook, IL, USA)
**Flexural**	
Test Type	Three-point bending
Span length	52 mm
Strain rate	10 mm/min
Standard	ASTM D790
Device	Imada MX2 (Northbrook, IL, USA)
**Impact**	
Test Type	Charpy
Samples	Notched
Release height	367 mm
Standard	ASTM D6110
Device	Terco MT 220 (Kungens Kurva, Sweden)
**Microhardness**	
Method	Vickers
Applied load	200 gF
Indentations’ duration	10 s
Standard	ASTM E384—17
Device	300 Innova Test (Maastricht, The Netherlands)

## Data Availability

The data presented in this study are available upon request from the corresponding author.
